# Shifts in the swine nasal microbiota following *Bordetella bronchiseptica* challenge in a longitudinal study

**DOI:** 10.3389/fmicb.2023.1260465

**Published:** 2023-09-29

**Authors:** Daniel W. Nielsen, Samantha J. Hau, Kathy T. Mou, David P. Alt, Susan L. Brockmeier

**Affiliations:** ^1^National Animal Disease Center, USDA Agricultural Research Service, Ames, IA, United States; ^2^Oak Ridge Institute for Science and Education (ORISE), Oak Ridge Associated Universities (ORAU), Oak Ridge, TN, United States

**Keywords:** swine, nasal, upper respiratory tract (URT), microbiota, *Bordetella bronchiseptica*, post-weaning

## Abstract

*Bordetella bronchiseptica* is a widespread, highly infectious bacterial pathogen that causes respiratory disease in swine and increases the severity of respiratory infections caused by other viral or bacterial pathogens. However, the impact of *B. bronchiseptica* infection on the swine respiratory microbiota has not been thoroughly investigated. Here, we aim to assess the influence of *B. bronchiseptica* infection on the community structure and abundance of members of the swine nasal microbiota. To do so, the nasal microbiota of a non-infected control group and a group infected with *B. bronchiseptica* (BB group) were characterized prior to *B. bronchiseptica* strain KM22 challenge (day 0) and on selected days in the weeks following *B. bronchiseptica* challenge (days 1, 3, 7, 10, 14, 21, 36, and 42). *Bordetella bronchiseptica* was cultured from nasal samples of the BB group to assess nasal colonization. The results showed that *B. bronchiseptica* colonization did not persistently affect the nasal bacterial diversity of either of the treatment groups (alpha diversity). However, the bacterial community structures (beta diversity) of the two treatment groups significantly diverged on day 7 when peak colonization levels of *B. bronchiseptica* were detected. This divergence continued through the last sampling time point. In addition, *Pasteurella, Pasteurellaceae* (unclassified), *Mycoplasma, Actinobacillus, Streptococcus, Escherichia-Shigella*, and *Prevotellaceae* (unclassified) showed increased abundances in the BB group relative to the control group at various time points. This study revealed that *B. bronchiseptica* colonization can disturb the upper respiratory tract microbiota, and further research is warranted to assess how these disturbances can impact susceptibility to secondary infections by other respiratory pathogens.

## Introduction

*Bordetella bronchiseptica* is one of the several bacterial respiratory pathogens that is associated with the costly multifactorial swine respiratory syndrome, porcine respiratory disease complex (PRDC) (Opriessnig et al., 2011). This organism is highly prevalent in swine and is commonly recovered from swine respiratory tracts (Brockmeier et al., 2012). *Bordetella bronchiseptica* colonizes the nasal cavity, trachea, and lung and is easily transmitted by direct contact or aerosol droplets. The bacteria cause pneumonia and atrophic rhinitis with clinical signs typically appearing 2–3 days after infection (Brockmeier et al., 2012). Clinical signs are more severe depending on age, immune status, and coinfection with other agents (Brockmeier et al., 2012). Although clinical signs may abate several weeks following initial infection, *B. bronchiseptica* may continue to colonize for months (Brockmeier et al., 2012).

*Bordetella bronchiseptica* also plays an important role in enhancing colonization and disease of important bacterial agents such as *Actinobacillus pleuropneumoniae, Glaesserella parasuis, Pasteurella multocida*, and *Streptococcus suis*, which are pathogens, potential pathogens, or pathobionts associated with PRDC (Harris and Switzer, 1968; Vecht et al., 1989, 1992; Dugal et al., 1992; Brockmeier et al., 2001; Brockmeier, 2004; Brockmeier and Register, 2007; Loera-Muro et al., 2016). *Bordetella bronchiseptica* predisposes animals to disease by *S. suis* (Vecht et al., 1989, 1992), allows *P. multocida* to colonize and contribute to disease (Harris and Switzer, 1968; Dugal et al., 1992; Brockmeier et al., 2001; Brockmeier and Register, 2007), promotes colonization of the upper respiratory tract (URT) with *G. parasuis* (Brockmeier, 2004), and, as shown in *in vitro* biofilm assays, may provide a niche environment for *A. pleuropneumoniae* to survive in the host URT (Loera-Muro et al., 2016).

In pigs, the composition and diversity of the respiratory tract microbiome may be important in preventing PRDC (Niederwerder, 2017; Pirolo et al., 2023). The URT microbiome is of particular interest as the URT is a primary reservoir for pathogens that reach the lungs (Pirolo et al., 2023). Differences in the microbial community of the URT have been identified for animals displaying clinical PRDC when compared with healthy counterparts, including differences in beta diversity measures and the prevalence of genera containing respiratory pathogens such as *Pasteurellaceae* (unclassified), *Actinobacillus*, and *Streptococcus* (Rampelotto et al., 2022). Changes in the microbial community of the URT have also been indicated for certain PRDC bacterial agents as low species richness and diversity in the URT have been associated with the development of Glässer's disease (Correa-Fiz et al., 2016). The composition and diversity of the URT microbiota may also be impacted by *B. bronchiseptica* infection; however, the relationship between *B. bronchiseptica* and the swine URT microbial population has not been thoroughly investigated. When defining the normal inhabitants of the URT, Pirolo et al. (2023) found that the genus *Bordetella* was more abundant in the posterior nasal cavity compared with the tonsils of swine, while *S. suis* was more abundant in the tonsils (Pirolo et al., 2023). In mice, *B. bronchiseptica* (strain RB50) has also been shown to displace the culturable host microbiota from the nasal cavity (Weyrich et al., 2014).

The findings of previous studies led us to hypothesize that infection with *B. bronchiseptica* could disturb the equilibrium of the microbiota of the nasal cavity and may influence the abundance of genera associated with PRDC. In this study, we characterized and compared the nasal microbiota of pigs inoculated with *B. bronchiseptica* with non-inoculated pigs over a 42-day period using 16S rRNA sequencing. We identified a divergence in the nasal microbiota communities during *B. bronchiseptica* infection starting on day 7 compared with the control group (beta diversity). No consistent changes were noted within the two groups (alpha diversity). Genera associated with PRDC showed significant differences in abundance when *B. bronchiseptica* colonization levels either peaked or declined.

## Materials and methods

### Animal study design and sample collection

Twenty weaned, unvaccinated, 3-week-old, crossbred pigs were acquired. Pigs were randomly distributed into two ABSL2 rooms with 10 pigs in each room. Pigs were acclimated 12 days prior to the beginning of the study. Nasal swabs were collected from all pigs prior to the start of the study to ensure the absence of *B. bronchiseptica*. On day 0 of the experiment, pigs in the *B. bronchiseptica* (BB) group were inoculated intranasally with 1 ml (0.5 ml/nostril) PBS containing 10^6^ colony forming units (CFU)/ml of *B. bronchiseptica* strain KM22 (Nicholson et al., 2012). The second group of pigs (control group) were not inoculated with KM22, but they were sham inoculated with 2 ml (1 ml per nostril) of PBS. All pigs were evaluated daily for the presence of clinical signs of respiratory disease. The nasal cavity of all pigs was sampled on day 0 prior to inoculation and on the following days after inoculation: 1, 3, 7, 10, 14, 21, 36, and 42. On day 42, all pigs were humanely euthanized and necropsied to assess gross lesions.

Samples of the nasal microbiota were obtained via nasal wash followed by nasal swabbing. Sterile PBS (5 ml) was applied via a syringe into the nostrils, and effluent PBS was collected. FLOQSwabs (Copan Flock Technologies, Murrieta, CA) were inserted into each nostril and placed in the collected nasal wash. Nasal samples were vortexed, and the nasal swab was removed prior to centrifugation at 10,000 rpm for 10 min at 4°C. The supernatant was decanted, and the pellet was resuspended in 200 μl of PBS. Samples were stored in a 96-well plate at −80°C for subsequent DNA extraction.

*Bordetella bronchiseptica* colonization was determined by plating serial 10-fold dilutions of post-challenge (days 1, 3, 7, 10, 14, 21, 36, and 42) nasal wash on selective blood agar plates containing 20 μg/ml penicillin, 10 μg/ml amphotericin B, 10 μg/ml streptomycin, and 10 μg/ml spectinomycin (Brockmeier et al., 2002; Brockmeier, 2004; Nicholson et al., 2009). Plates were incubated at 37°C with 5% CO_2_ for 2 days and were subsequently enumerated for bacterial quantification. The log_10_ CFUs were plotted using GraphPad Prism (Dotmatics, San Diego, CA).

### DNA extraction, amplification, and sequencing

Bacterial DNA from nasal samples was extracted using the PowerMag Microbiome DNA/RNA Isolation Kit (Qiagen, Hilden, Germany) and the epMotion 5075 workstation (Eppendorf, Hamburg, Germany), following the manufacturer's instructions. DNA concentration was assessed with the Quant-IT PicoGreen dsDNA Kit (Thermo Fisher Scientific, Waltham, MA). All samples were processed through the MiSeq Wet Lab SOP to prepare the hypervariable V4 region of 16S rRNA gene sequences for Illumina MiSeq sequencing (Kozich et al., 2013): https://github.com/SchlossLab/MiSeq_WetLab_SOP/blob/master/MiSeq_WetLab_SOP.md. Mock community and negative extraction controls were also included for sequencing (Allen et al., 2016). Once samples were processed, pooled, and normalized to at least 1 nM, they were submitted to the NADC Genomics Facility in Ames, IA for preparation of 250 bp paired-end library and sequencing on the MiSeq instrument (Illumina, Inc. San Diego, CA) using version 2 chemistry.

### Sequence processing and taxonomy assignment

Raw FASTQ data were retrieved from the MiSeq platform and processed using mothur (version 1.48.0) and MiSeq SOP (https://mothur.org/wiki/miseq_sop/, accessed 12/13/2022). Sequences were assigned taxonomies with the SILVA release 132 database (https://www.arb-silva.de/) and identified a total of 1,889 operational taxonomic units (OTUs). After processing sequences and subsampling samples to 7,395 sequences with mothur, there were 1,636 OTUs. The number of samples per day is presented in Table 1. Two samples were excluded due to poor sequencing depth, and two samples were inadvertently contaminated during sample collection.

**Table 1:** Nasal samples from each treatment group and time point used in the analyses.

**Day**	**BB**	**Control**
D0	10	10
D1	9	10
D3	9	10
D7	8	10
D10	10	10
D14	10	10
D21	10	10
D36	10	10
D42	10	10

BB, Bordetella bronchiseptica.

### Nasal microbiota data and statistical analyses

Data analysis and figure generation were conducted using R version 4.1.2 (R Core Team, 2021). The R packages ggplot2 (Wickham et al., 2016) and cowplot (Wilke et al., 2019) were used to visualize data. The R packages such as vegan (Oksanen et al., 2022), phyloseq (McMurdie and Holmes, 2013), philentropy (Drost, 2018), and tidyverse (Wickham et al., 2019) were used to analyze various alpha (Shannon and Inverse Simpson) and beta (Bray–Curtis dissimilarity matrices based on OTU abundances) diversity measures of the samples from each treatment group (BB and control) and day. To assess for any significant differences in alpha and beta diversities between treatment groups on a given day (*p* ≤ 0.05), Wilcoxon rank-sum test and permutational multivariate analysis of variance using distance matrices (adonis) function (Oksanen et al., 2007) were conducted on the paired data, respectively. The average number of distinct OTUs per day for each treatment was calculated, and a two-way ANOVA was performed using GraphPad Prism.

Raw data (non-subsampled data with singletons removed) were filtered to remove samples that had fewer than 7,394 sequences and OTUs that occurred <10 times globally. To identify differentially abundant OTUs between the groups, the DESeq2 package version 1.34.0 (Anders and Huber, 2010; Love et al., 2014) (open source, www.bioconductor.org) was used. OTUs were combined at the genus level, and the Benjamini–Hochberg adjustment was used to limit the false discovery rate of contrasts. The significance of the log_2_-fold change of comparisons was determined using the Wald test and a cutoff of *p* ≤ 0.05. OTUs that were differentially abundant (log_2_-fold changes with adjusted *p*-values ≤ 0.05) and showed no significant differences between the two groups on day 0 were selected for further study. Genera associated with porcine respiratory disease complex were also examined even if they were significantly different on day 0, to observe if changes in abundance occurred.

## Results

### *Bordetella bronchiseptica* colonization

**Figure 1:**
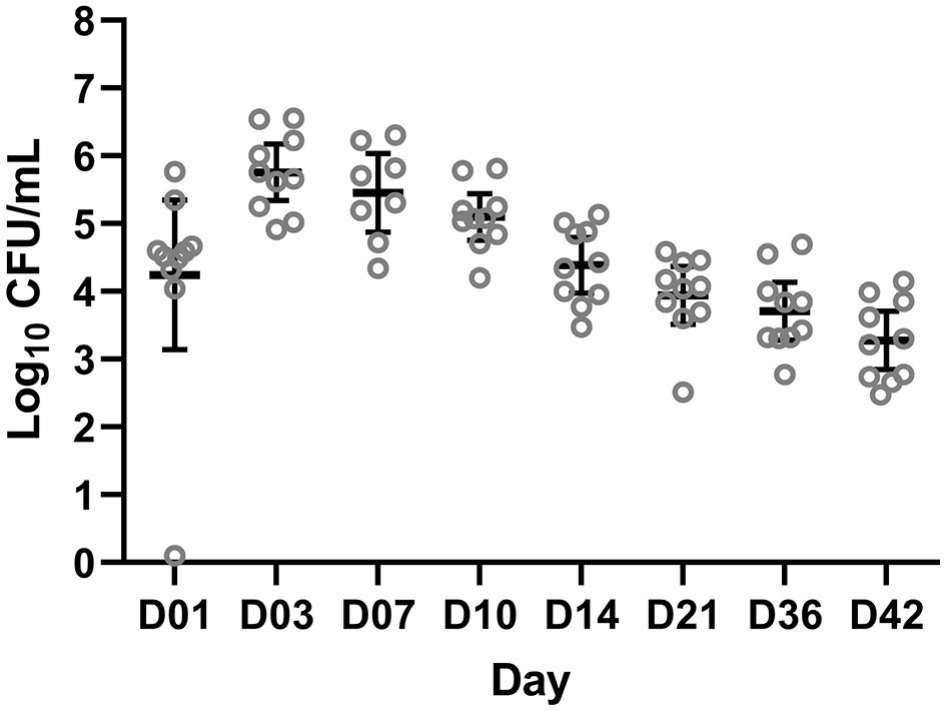
Colony forming units (CFUs) of *Bordetella bronchiseptica* quantified from the BB group on a log_10_ scale (*y*-axis) for each sampling day (*x*-axis) post-challenge. Each gray circle represents a different animal sample. The mean and 95% confidence intervals are represented by the black bars.

After the challenge, animals were monitored for clinical signs of *B. bronchiseptica* infection twice daily. No animals developed respiratory clinical signs. One animal had a 1 cm consolidated patch in the left middle lung lobe consistent with *B. bronchiseptica* bronchopneumonia at necropsy on day 42. Colonization of *B. bronchiseptica* was detected in all pigs of the BB group on day 1. Average colonization peaked on days 3 and 7 at 5.74 × 10^5^ and 2.86 × 10^5^ log_10_ CFU/ml, respectively (Figure 1).

### Community and diversity changes in the nasal microbiota after *Bordetella bronchiseptica* infection

**Figure 2:**
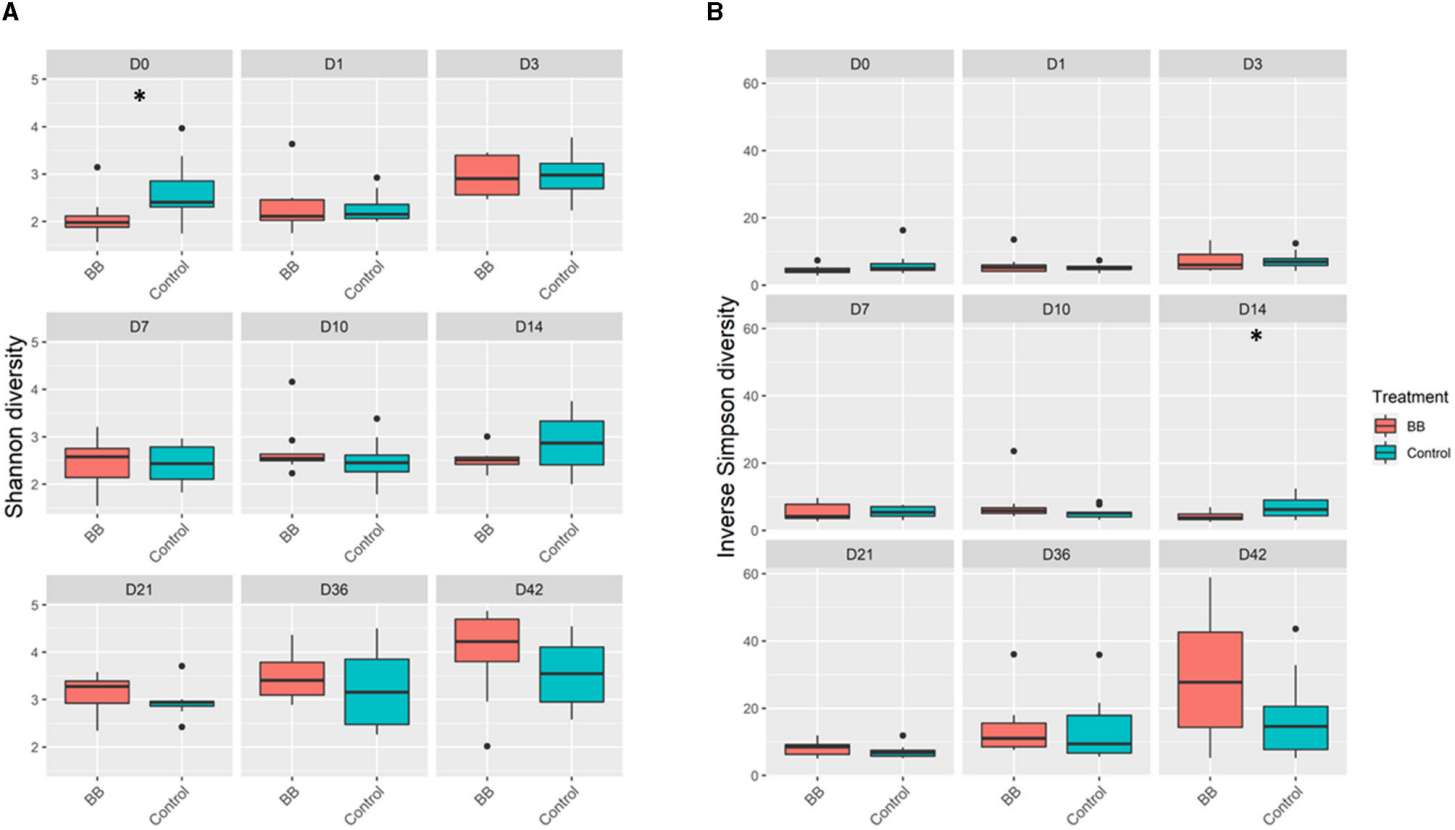
Alpha diversity comparisons between the BB and control groups. BB (red) had significantly different (*) Shannon diversity relative to control (blue) only on day 0 (D0) relative to the control (blue) **(A)**. Significant differences in inverse Simpson diversity relative to control (blue) were observed only on day 14 (D14) **(B)**. Pairwise comparisons within groups across time and between groups at each time point were performed using the Wilcoxon rank-sum test at a significance level of 0.05 (*p* ≤ 0.05*).

Community richness was compared between the control and the *B. bronchiseptica* (BB) groups, and no significant and persistent changes were observed in the study. The Shannon diversity index demonstrated statistical significance only on day 0 (*p* = 0.029; Figure 2A, Supplementary Table S1). However, this difference was not observed in the inverse Simpson diversity index (Figure 2B, Supplementary Table S1). The inverse Simpson diversity index demonstrated a significant difference between the control and BB groups on day 14 (*p* = 0.035) (Figure 2B, Supplementary Table S1). When the mean number of distinct OTUs per day for each treatment group was calculated, no statistical significance was observed between the BB and control groups (Supplementary Figure S1). However, there was an upward trend toward a higher number of distinct OTUs per sample as the study progressed.

**Figure 3:**
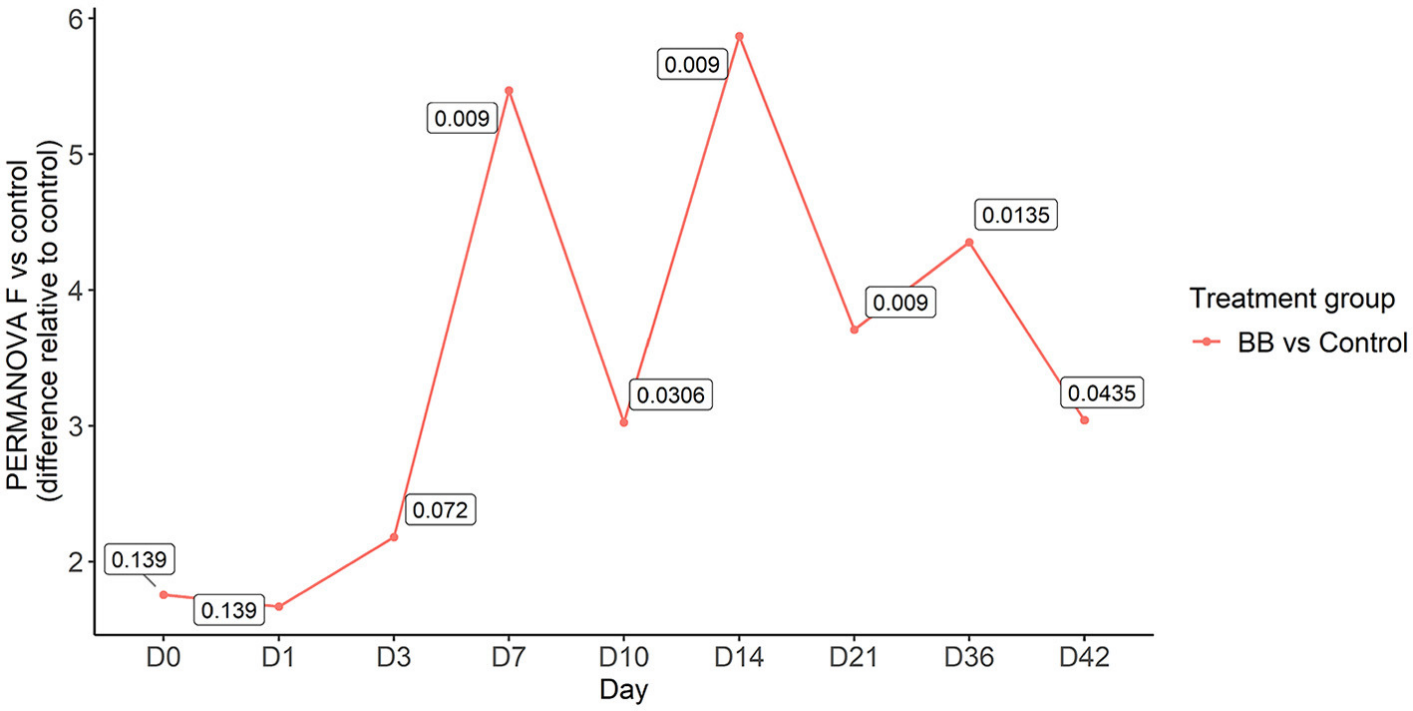
PERMANOVA *F*-statistic of nasal bacterial community analysis of the BB group relative to the control group over the 42-day period. Each point represents the PERMANOVA test statistic (F: intergroup dissimilarity/intragroup dissimilarity) of the BB group over time (days 0 to 42), with control group set as 0 at the baseline. *p*-values are contained within the text boxes; days 7–42 are considered statistically significant.

**Figure 4:**
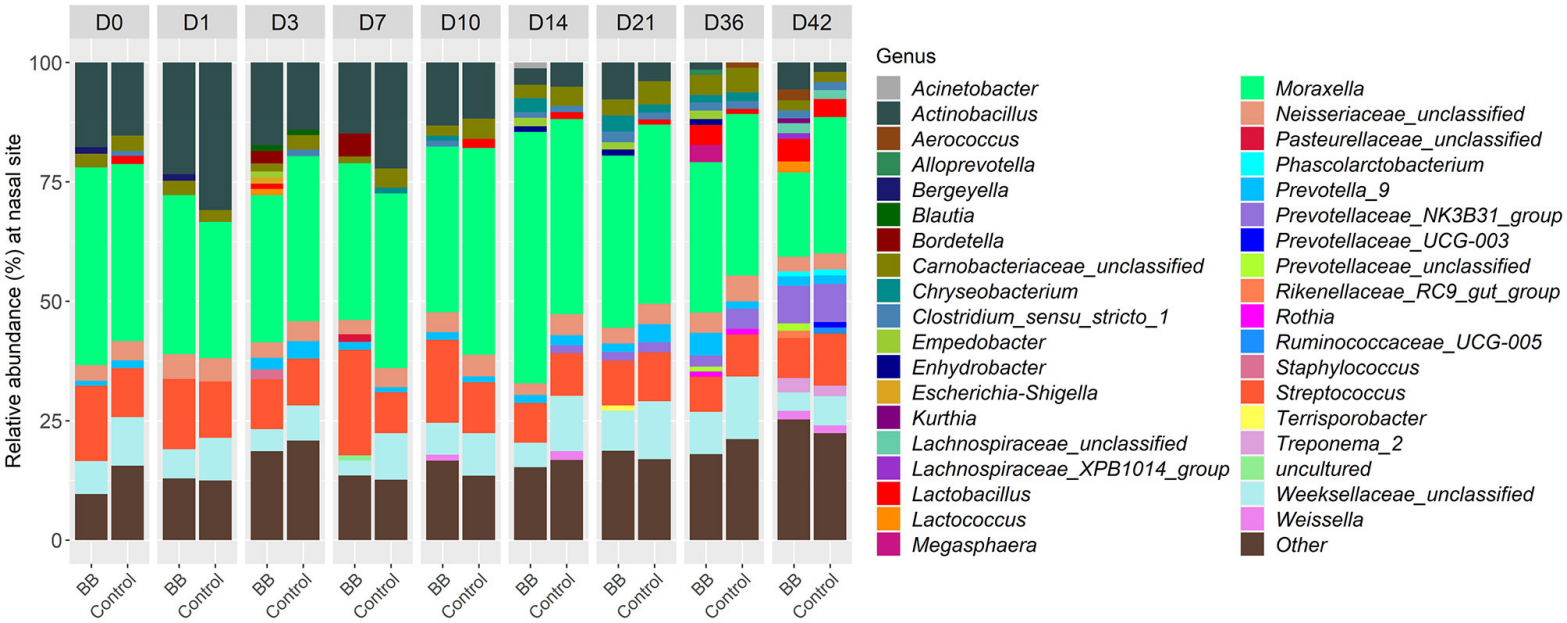
Relative abundances of nasal bacterial genera over the 42-day period between the BB and control groups. Within each day (day numbers listed at the top of the bar plots), each bar plot shows the percentages of total genera found in each group. Only genera with more than 1% abundance are shown. Genera with <1% abundance are grouped in the “Other” category.

To assess the changes in the bacterial community structure between BB and control groups on a given day, a PERMANOVA pairwise comparison test was performed using the treatment-by-day variable using metaMDS from the vegan package (Supplementary Figure S2). In addition, the PERMANOVA *F*-statistic was plotted against time to assess the magnitude of change in the community structure of BB relative to control (Figure 3). After the challenge, no significant changes were observed on days 1 and 3 for the BB group when compared with the control group. However, significant changes were observed on the other sampling days 7–42 (Figure 3). On day 7, the *F*-statistic rose to 5.47, and it peaked on day 14 (5.87). The nasal microbial communities are presented in Figure 4, which depicts the relative abundance of genera present in the nasal cavity by treatment and day. The changes from days 7 to 42 suggest that the nasal bacterial community remains dissimilar from the control group persistently (Figure 3).

Variables such as treatment, day, and treatment by day were examined with the adonis function to determine the level each variable contributed to the observed shifts in the nasal community between the BB and control groups (Table 2). The treatment, day, and treatment-by-day variables exhibited significantly strong effects (*p* ≤ 0.0001). The day of sampling was the strongest contributor to the variation observed in the nasal microbial community between the two groups as the day variable had the largest *R*-square value (*R*^2^ = 0.367), followed by treatment by day (*R*^2^ = 0.075) and finally treatment (*R*^2^ = 0.028). Therefore, treatment was significant, but time appeared to have the greatest impact.

**Table 2:** Impact of treatment, day, and treatment by day on microbiome changes.

**Variable**	***R* ^2^**	**p-value**
Treatment	0.028	1.0E-04
Day	0.367	1.0E-04
Treatment by day	0.075	1.0E-04

The treatment, day, and treatment by day were examined with the Adonis function to assess how each variable contributed to the observed shifts in the nasal community composition between the BB and control groups.

### Genera in the nasal cavity that were differentially abundant in response to *B. bronchiseptica* colonization

**Figure 5:**
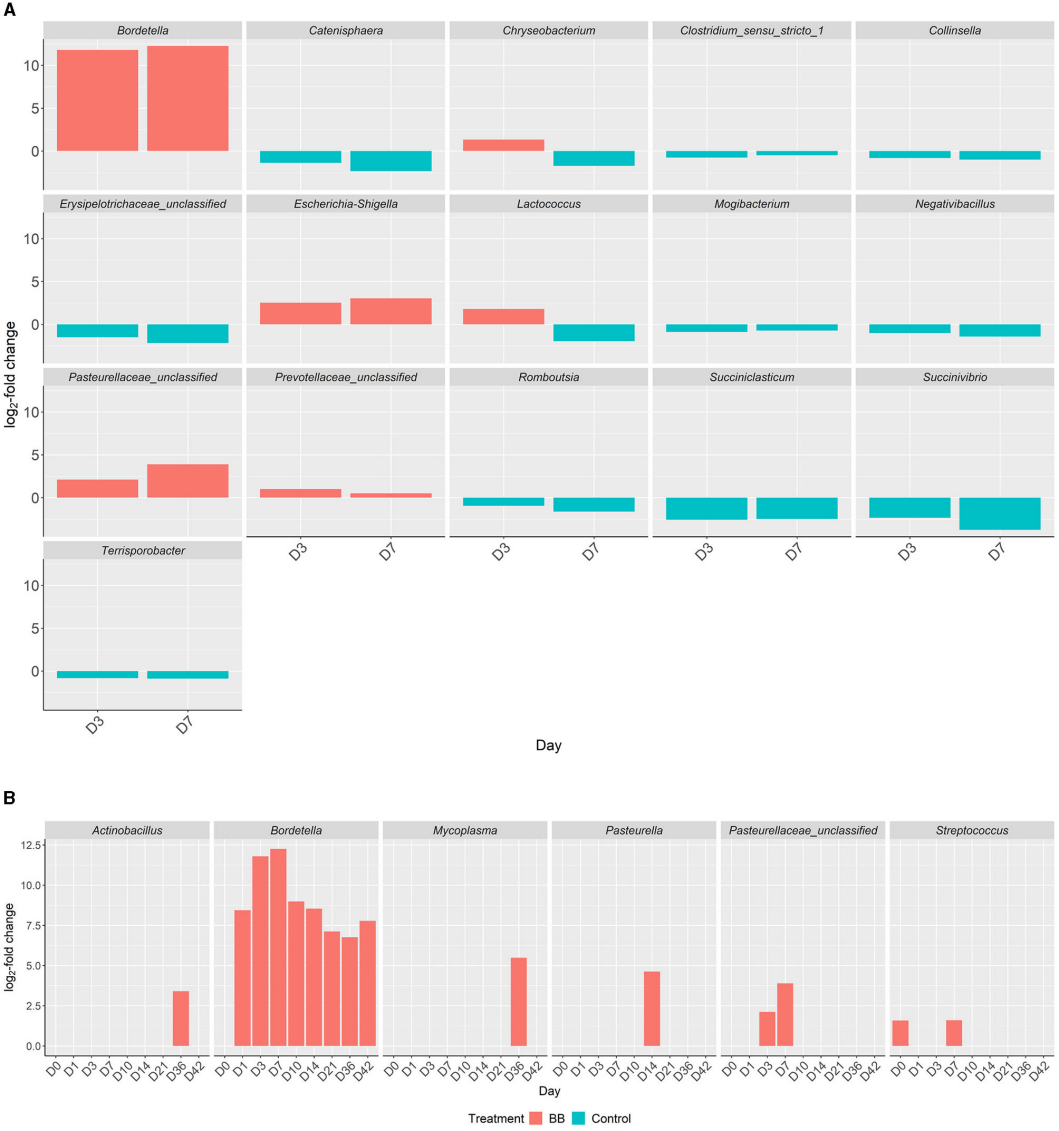
Differential abundances of genera in the respiratory tract between the BB and control groups. Genera with significant increased abundances between the treatment groups, BB (red) and control (blue), during peak *Bordetella* colonization, days 3 and 7 **(A)**. *X*-axis represents the day, *y*-axis displays the log_2_-fold changes of the genera, and the corresponding genus is listed above each panel. Genera with significant increased abundances between treatment groups, BB (red) and control (blue), associated with PRDC **(B)**, even if statistically significant abundances are present on day 0.

Between the BB and control groups, 147 genera representing 150 OTUs were differentially abundant across the time course of the study when the 49 genera different on day 0 were excluded. Although there were 196 differentially expressed genera (Supplementary Table S2), only 37 genera had >1% relative abundance per day over the course of the study (Figure 4). As expected, *Bordetella* was differentially more abundant in the BB group than in the control group (Figures 4, 5A, B). However, no other genera were persistently differentially abundant for the following days 1, 3, 7, 10, 14, 21, 36, and 42 when the BB group and control group were compared (data not shown). No consistent genera changes were noted, suggesting that differences in community structure were associated with differential abundances of genera between the groups on different days.

Although differentially abundant for the entire study between the BB and control groups, the genus *Bordetella* comprised >1% of the BB group only on the days associated with peak colonization, days 3 and 7 (2.57 and 4.78%, respectively; shown in Figures 1, 4). In addition to *Bordetella*, other bacterial genera were also differentially abundant on days 3 and 7. *Escherichia-Shigella, Pasteurellaceae* (unclassified), and *Prevotellaceae* (unclassified) were more abundant in the BB group compared with the control. Alternatively, *Catenisphaera, Clostridium sensu stricto 1, Collinsella, Erysipelotrichaceae* (unclassified), *Mogibacterium, Negativibacillus, Romboutsia, Succiniclasticum, Succinivibrio*, and *Terrisporobacter* showed increased abundance in the control group compared with the BB group (Figure 5A; Supplementary Table S2).

Interestingly, certain genera showed increased abundances later in the study. On days 14–42, *Micrococcaceae* (unclassified), *Pseudochrobactrum*, and *Stenotrophomonas* showed increased abundance in the BB group compared with the control group (Supplementary Table S2). On days 10–36, *Enterorhabdus* showed increased abundance in the control group compared with the BB group; however, this trend was not observed on day 42 (Supplementary Table S2).

### PRDC-associated genera that were differentially abundant in response to *B. bronchiseptica* colonization

When the abundances of genera associated with PRDC were examined, *Actinobacillus* and *Mycoplasma* were significantly more abundant in the BB group than in the control group on day 36 (Figure 5B). *Pasteurella* was significantly more abundant in the BB group than in the control group on day 14 (Figure 5B). OTUs identified as *Pasteurellaceae* (unclassified), which may encompass *G. parasuis*, were significantly more abundant in the BB group on days 3 and 7. The differential abundance of *Pasteurellaceae* (unclassified) corresponded to the peak colonization of *B. bronchiseptica* (Figures 1, 5B). Although the *Streptococcus* genus was significantly more abundant on day 0 for the BB group compared with the control group, *Streptococcus* was not statistically different on days 1 and 3, but the genus peaked on day 7 for the BB group (Figure 5B). There were no significant differences observed for *Trueperella* or *Salmonella*.

## Discussion

*Bordetella bronchiseptica* is a primary respiratory pathogen of pigs that is known to impact the severity of respiratory infection by increasing inflammation and enhancing colonization with other bacteria that contribute to PRDC (Opriessnig et al., 2011; Brockmeier et al., 2012). Though many studies have investigated *B. bronchiseptica* monoinfection and coinfections (Brockmeier, 2004; Nicholson et al., 2009, 2012), a few studies have been conducted evaluating the impact of *B. bronchiseptica* on the URT microbiota. There is evidence to indicate that *B. bronchiseptica* infection could disrupt the normal microbiota as it has been shown to enhance colonization with other bacterial respiratory pathogens in pigs and displace the culturable microbiota in the nasal cavity of mice (Brockmeier, 2004; Opriessnig et al., 2011; Weyrich et al., 2014). This current study was a longitudinal evaluation of the nasal microbiota of pigs following *B. bronchiseptica* infection to assess the changes *B. bronchiseptica* causes to the nasal microbiota and increase our understanding of bacterial interactions in the nasal cavity that may contribute to increases in secondary bacterial infections.

In this study, we observed that *B. bronchiseptica* colonization peaked on days 3 and 7, and the bacteria were detected at the end of the study (day 42). These results correspond to previous studies, which found that *B. bronchiseptica* strain KM22 colonization peaked in the nasal cavity on day 7 post-infection in swine and remained detectable until the end of that study on day 57 (Nicholson et al., 2012). In contrast to previous studies on *B. bronchiseptica* (Nicholson et al., 2009, 2012), we did not observe clinical signs, and minimal lesions were detected at necropsy on day 42. The differences in clinical disease and lesion severity may be due to weaning age and the age of inoculation. In this study, we used animals weaned at 3 weeks of age and inoculated them at ~5 weeks of age. In traditional *B. bronchiseptica* studies, pigs are early weaned and inoculated ~1 week of age (Nicholson et al., 2009, 2012), which enhances the severity of *B. bronchiseptica* disease. However, pigs can be exposed at weaning when co-mingled, which is consistent with the model used in this study (Brockmeier et al., 2012). The microbiota evaluation found that the challenge with *B. bronchiseptica* did not result in a significant reduction of unique OTUs and caused no persistent changes in richness as indicated by Shannon and Inverse Simpson diversity indices. This was similar to the findings by Rampelotto et al. (2022), where no differences were found in alpha diversity between pigs showing signs of PRDC and asymptomatic animals, although PRDC was defined clinically, and causative agents were not identified. In contrast to our findings, Correa-Fiz et al. (2016) found significant differences in the Shannon diversity index between farms with Glässer's disease compared with healthy farms. However, their study was observational among different farms and investigated *G. parasuis*, a different etiological agent of PRDC.

Interestingly, *B. bronchiseptica* challenge resulted in persistent changes in the nasal bacterial community structure from days 7 to 42 as indicated by beta diversity (Bray–Curtis) measurements, suggesting that the nasal bacterial community was unable to recover following the introduction of *B. bronchiseptica*. Previous studies have examined the relationship between species of clinical interest and *B. bronchiseptica* through various approaches. Brockmeier (2004) found that *B. bronchiseptica* increased colonization of the upper respiratory tract with *G. parasuis*. Votsch et al. (2021) found that pre-infection with *B. bronchiseptica* promoted adherence and colonization by *S. suis* in a porcine precision-cut long slice model. Zhao et al. (2011) found that *B. bronchiseptica* was isolated from 18.6% of porcine lung samples collected over a 6-year period from provinces in central and eastern China, and *S. suis, G. parasuis*, and *Escherichia coli* were isolated most frequently in association with *B. bronchiseptica* (Zhao et al., 2011). Previous studies have also found that pigs infected with *B. bronchiseptica* may be more vulnerable to colonization by *P. multocida* (Chanter et al., 1989; Brockmeier et al., 2001). Although not species-specific, our study concurs with previous studies, as we observed increases in the abundance of *Streptococcus* (day 7), *Escherichia*-*Shigella* (days 3 and 7), *Pasteurella* (day 14), and *Pasteurellaceae* (unclassified), which would likely encompass the genus *Glaesserella* (days 3 and 7), for the BB group compared with the control group during peak colonization.

Rampelotto et al. (2022) also found significant differences in beta diversity between the URT of symptomatic and asymptomatic pigs of respiratory disease at weaning and end of the nursery phases (Rampelotto et al., 2022). *Actinobacillus, Streptococcus, Porphyromonas, Veillonella*, and *Pasteurellaceae* (unclassified) were more abundant in pigs with clinical signs at weaning and end of the nursery phases compared with pigs without clinical signs (Rampelotto et al., 2022). *Mycoplasma* was prevalent in both symptomatic and asymptomatic pigs at weaning, but Rampelotto et al. (2022) observed lower abundance at later time points. In this study, we also found differences in the abundance in several of these genera as *Actinobacillus* (day 36), *Streptococcus* (days 0 and 7), and *Pasteurellaceae* (unclassified; days 3 and 7) were significantly more abundant in the BB group than in the control group (Supplementary Table S2 and Figure 5B). We also found that *Mycoplasma* (day 36) was significantly more abundant in the BB group than in the control group (Supplementary Table S2 and Figure 5B). However, this study did not find consistent results for the genus *Veillonella* among the BB and control groups (Supplementary Table S2). A Previous study by Correa-Fiz et al. (2016) found that *Actinobacillus, Corynebacteria, Planobacterium, Pseudoflavonifractor, Staphylococcus, Streptococcus, Haemophilus* (which would include *Glaesserella*, a member of the *Pasteurellaceae* family), *Kingella, Moraxella*, and *Mycoplasma* were more abundant in diseased animals from a farm with Glässer's disease (caused by *G. parasuis*) (Correa-Fiz et al., 2016). Although our current study focused on the challenge with *B. bronchiseptica, Actinobacillus* (day 36), *Corynebacterium 1* (days 0, 7, 10, 14, 21, 36, and 42), *Staphylococcus* (days 21 and 36), *Streptococcus* (days 0 and 7), *Pasteurellaceae* (unclassified; days 3 and 7), and *Mycoplasma* (day 36) were significantly more abundant in the BB group than in the control group (Supplementary Table S2 and Figure 5B). Combined with previous studies, our results suggest that *Actinobacillus, Streptococcus*, and *Pasteurellaceae* (unclassified) may be more abundant in the nasal cavity when swine are infected with PRDC pathogens, which may contribute to complicated respiratory infections and/or the development of systemic disease.

In this study, we analyzed the data using OTUs instead of ASVs. However, previous studies observed that a high correlation was observed when using ASVs and OTUs for alpha diversity (Shannon) and beta diversity (Bray–Curtis) measurements (Chiarello et al., 2022), indicating that the impact of using OTUs instead of ASVs may be minimal. Additionally, we acknowledge that ASVs might have provided additional resolution to the species level, but species-level resolution presents additional challenges. For example, species-level resolution may not be adequate to determine whether any isolate of a species will result in disease or death of a host as intraspecies differences occur (MacInnes and Desrosiers, 1999; Brockmeier et al., 2014).

In conclusion, this study observed a significant change in the composition of the swine nasal microbial community between the BB and control groups beginning on day 7 and continuing through the last sampling time point. After the challenge, there were also significant changes observed in the abundance of nasal genera that can be attributed to the changing colonization levels of *B. bronchiseptica* in the BB group. Notably, we observed *Pasteurellaceae* (unclassified) was more abundant in the BB group than in the control group on days 3 and 7, during peak *Bordetella* colonization. The significance of these changes is unknown, highlighting the need for further investigation into the effect of such disturbances on the protective role of the URT microbiota and swine respiratory health.

## Data availability statement

The datasets presented in this study can be found in online repositories. The names of the repository/repositories and accession number(s) can be found at: https://www.ncbi.nlm.nih.gov/, PRJNA525911.

## Ethics statement

The animal study was approved by National Animal Disease Center Institutional Animal Care and Use Committee. The study was conducted in accordance with the local legislation and institutional requirements.

## Author contributions

DN: Methodology, Data curation, Formal analysis, Project administration, Validation, Visualization, Writing—original draft, Writing—review and editing. SH: Conceptualization, Formal analysis, Investigation, Methodology, Supervision, Writing—original draft, Writing—review and editing. KM: Data curation, Investigation, Methodology, Writing—review and editing. DA: Investigation, Methodology, Writing—review and editing. SB: Conceptualization, Investigation, Methodology, Project administration, Supervision, Writing—review and editing.

## Supplementary material

Supplementary Table S1 Pairwise Wilcoxon rank sum test between the BB and control groups on each day for the alpha diversity values (Shannon and Inverse Simpson; *p* ≤ 0.05^*^).

Supplementary Table S2 Relative log-fold changes of differentially abundant OTUs. Relative log-fold changes and corresponding operational taxonomic units (OTUs) were significantly and differentially abundant between the BB and control groups on any day as identified by the Wald test with a parametric fit in DESeq and Benjamini–Hochberg adjustment (padj).

Supplementary Figure S1 Mean number of distinct OTUs for each sample (*y*-axis) by day (*x*-axis) for the BB (red) and control (blue) groups. No statistical differences between the BB and control groups were found at any time point.

Supplementary Figure S2 Significant changes in the nasal microbial composition between the control and BB groups were observed on days 7, 10, 14, 21, 36, and 42. This non-metric multidimensional scaling (NMDS) ordination is another way of visualizing the data underlying the PERMANOVA results and is generated using the Bray–Curtis dissimilarity metric calculated with rarefied OTU abundance data (*k* = 2, stress = 0.169). The plot is split into nine treatment-by-day panels. The labels at the top of each panel refer to the day sampled. Day 0 samples were collected before the animals in the BB group were challenged. Each point represents one sample. The closer the samples are to each other, the more similar the microbial compositions of the samples are. Samples are linked to the treatment group centroid by segments, and the standard error of the treatment group is depicted with an ellipse. Within each day, the two groups are as follows: BB (red) and control (blue).
