# Supplementary figures and images for: Shifts in the swine nasal microbiota following Bordetella bronchiseptica challenge in a longitudinal study
Source: Front Microbiol. 2023 Sep 29;14:1260465. doi: 10.3389/fmicb.2023.1260465 (PMC10574184; doi:10.3389/fmicb.2023.1260465)

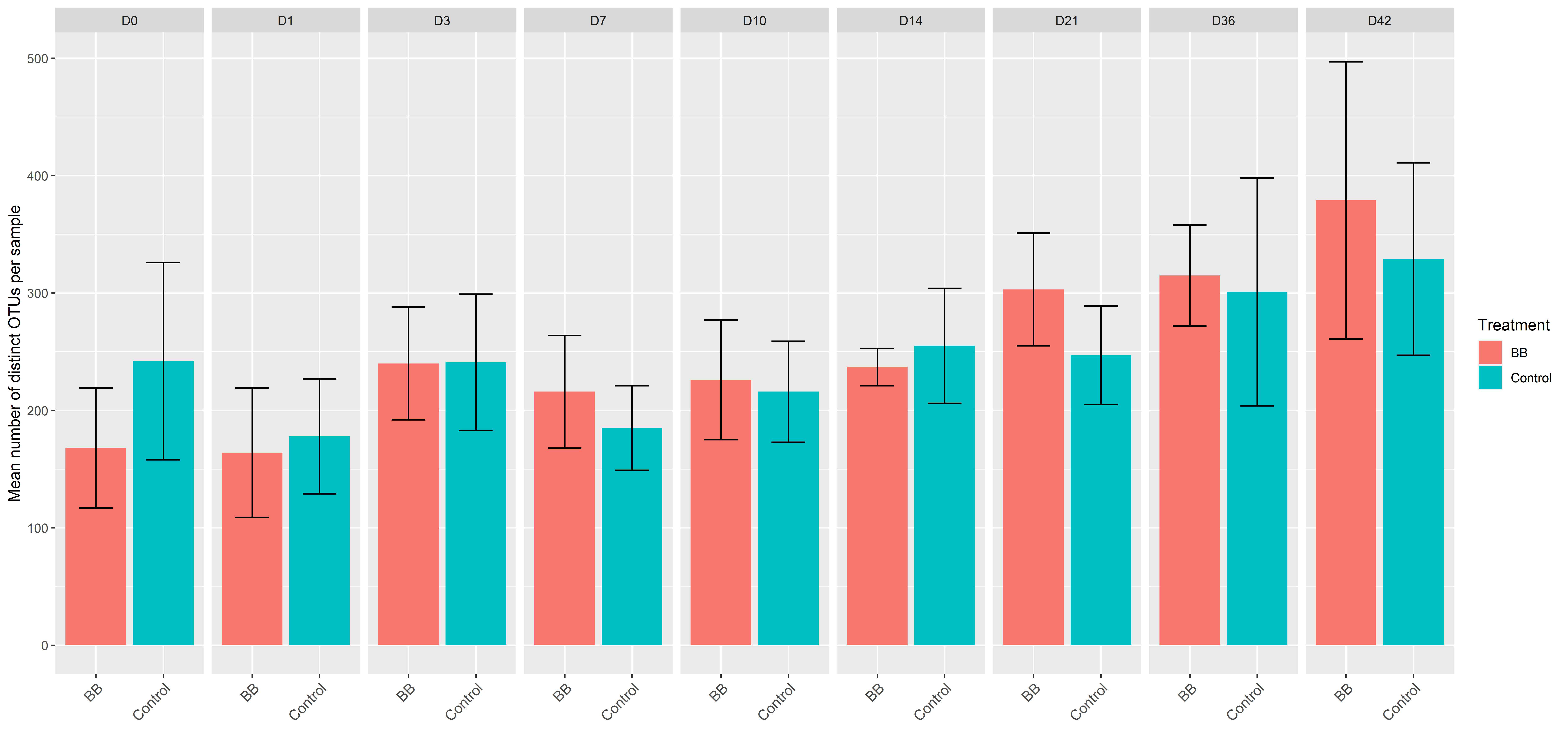

Supplement: Supplementary Figure S1 — Mean number of distinct OTUs for each sample (y-axis) by day (x-axis) for the BB (red) and control (blue) groups. No statistical differences between the BB and control groups were found at any time point. [file Image_1.TIF]

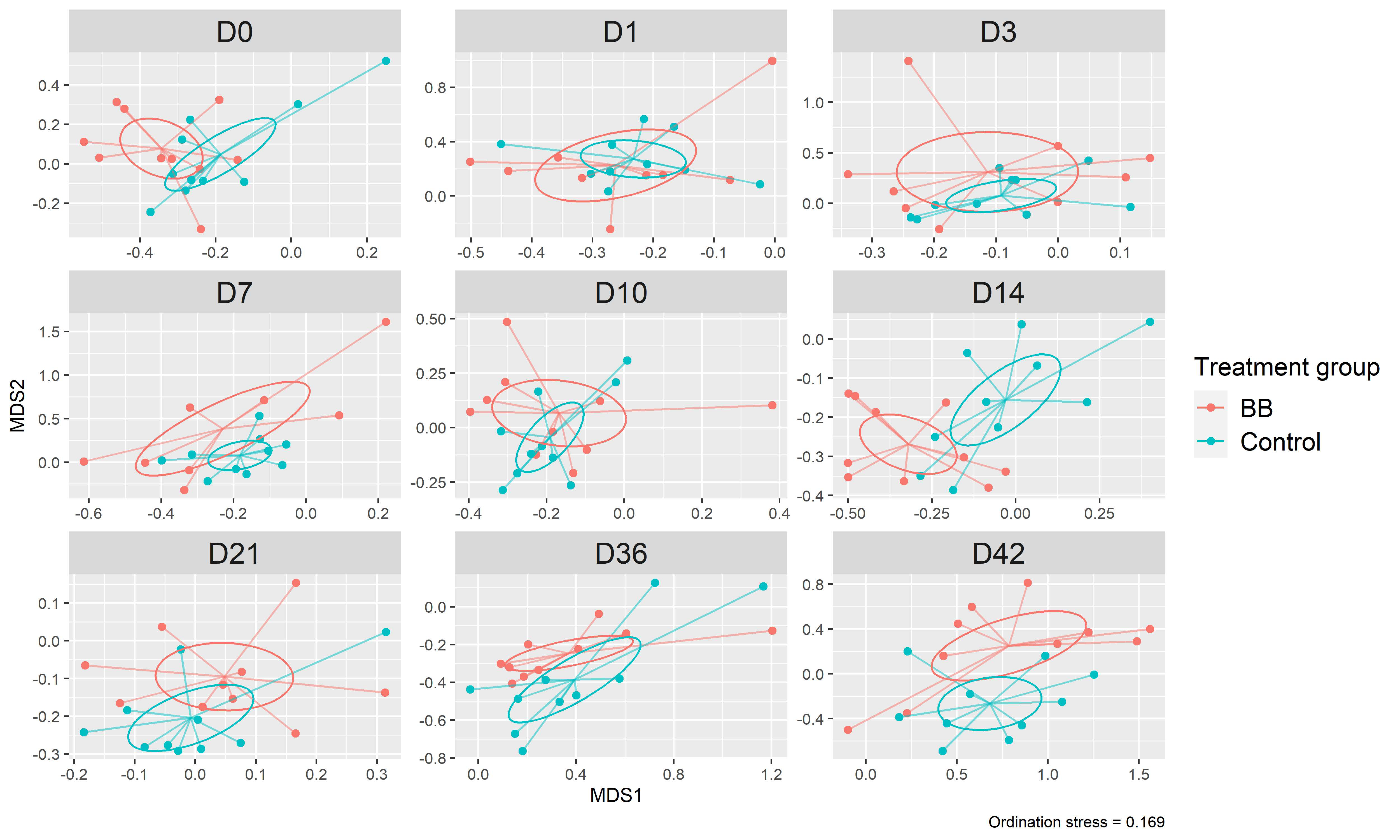

Supplement: Supplementary Figure S2 — Significant changes in the nasal microbial composition between the control and BB groups were observed on days 7, 10, 14, 21, 36, and 42. This non-metric multidimensional scaling (NMDS) ordination is another way of visualizing the data underlying the PERMANOVA results and is generated using the Bray–Curtis dissimilarity metric calculated with rarefied OTU abundance data (k = 2, stress = 0.169). The plot is split into nine treatment-by-day panels. The labels at the top of each panel refer to the day sampled. Day 0 samples were collected before the animals in the BB group were challenged. Each point represents one sample. The closer the samples are to each other, the more similar the microbial compositions of the samples are. Samples are linked to the treatment group centroid by segments, and the standard error of the treatment group is depicted with an ellipse. Within each day, the two groups are as follows: BB (red) and control (blue). [file Image_2.TIF]
